# The double burden of malnutrition in Indonesia: Social determinants and geographical variations

**DOI:** 10.1016/j.ssmph.2015.10.002

**Published:** 2015-11-18

**Authors:** Wulung Hanandita, Gindo Tampubolon

**Affiliations:** Cathie Marsh Institute for Social Research, University of Manchester, Humanities Bridgeford Street Building 2F, Oxford Road, Manchester M13 9PL, United Kingdom

**Keywords:** Double burden malnutrition, Underweight, Overweight, Indonesia, Social determinants, Multilevel model, Quantile regression

## Abstract

The presence of simultaneous under- and over-nutrition has been widely documented in low- and middle-income countries, but global nutritional research has seen only a few large-scale population studies from Indonesia. We investigate the social determinants as well as the geographical variations of under- and over-nutrition in Indonesia using the largest public health study ever conducted in the country, the National Basic Health Research 2007 (N=645,032). Multilevel multinomial logistic regression and quantile regression models are fitted to estimate the association between nutritional status and a number of socio-economic indicators at both the individual and district levels. We find that: (1) education and income reduce the odds of being underweight by 10–30% but at the same time increase those of overweight by 10–40%; (2) independent from the compositional effect of poverty, income inequality is detrimental to population health: a 0.1 increase in the Gini coefficient is associated with an 8–12% increase in the odds of an individual׳s being both under- and overweight; and (3) the effects that these determinants have upon nutritional status are not necessarily homogeneous along the continuum of body mass index. Equally important, our analysis reveals that there is substantial spatial clustering of areas with elevated risk of under- or over-nutrition across the 17,000-island archipelago. As of 2007, under-nutrition in Indonesia remains a ‘disease of poverty’, while over-nutrition is one of affluence. The income inequality accompanying Indonesia׳s economic growth may aggravate the dual burden of under- and over-nutrition. A more equitable economic policy and a policy that improves living standards may be effective for addressing the double burden.

## Highlights

• We study the double burden of malnutrition in Indonesia using multilevel model.
• Education and income protect Indonesians from being undernourished.
• At the same time, they also increase the likelihood of being overweight.
• Income inequality increases the odds of being both under- and overweight.
• The risk of dual malnutrition is spatially clustered within the islands of Indonesia.

## 1 Introduction

The simultaneous presence of under- and over-nutrition within populations of developing countries undergoing rapid economic transition has been widely documented (Gillespie and Haddad, 2003, Jehn and Brewis, 2009). The changes in dietary intake patterns and leisure-time activities associated with industrialisation and urbanisation are known to have contributed to an increased prevalence of obesity in numerous countries (Popkin, 1998, Popkin, 1999); at the same time, the problem of under-nutrition remains undefeated. This dual burden, which may also exist within a single household (Doak et al., 2005, Lee et al., 2012), is costly for the health as well as the economy of a nation. Under-nutrition impairs cognition (Sandjaja et al., 2013) and physical development (Mani, 2012), reduces economic productivity (Victora et al., 2008), raises the mortality rate, and even induces an intergenerational cycle of malnutrition (Barker, 1997); on the other extreme of the nutritional spectrum, over-nutrition is known to increase the risk of non-communicable diseases, inflate health care costs (Cawley and Meyerhoefer, 2012, Withrow and Alter, 2011), and reduce overall quality of life.

The body of nutritional epidemiology and development economics research suggests that, over and above the biological aspects of age and sex, socio-economic status, along with a number of ecological factors such as urban environment, area-level economic development and income inequality, seems to consistently determine the social distribution of malnutrition (Doak et al., 2005, Ha et al., 2011, Lee et al., 2012, Rahmanian et al., 2014, Roemling and Qaim, 2013, Shafique et al., 2007, Subramanian et al., 2007, Vaezghasemi et al., 2014). Notwithstanding the increasing number of studies in this stream of research, the literature, however, does not yet include sufficient evidence from Indonesia, which is the most populous developing country after China and India. To date, empirical evidence tends to come from South Asia, Africa and Latin America (see for example Corsi, Finlay, & Subramanian, 2011 or Jehn & Brewis, 2009). Little is known about the double burden of malnutrition in Indonesia, despite the fact that it is in a state of rapid economic and epidemiologic transition where industrialisation, urbanisation and political decentralisation are met with rising income inequality, widening regional disparities and a diminishing rate of poverty reduction (World Bank, 2014). All existing studies focusing on Indonesia (Doak et al., 2005, Oddo et al., 2012, Roemling and Qaim, 2013, Vaezghasemi et al., 2014, Winkvist et al., 2000) have thus far (1) dealt specifically with the coexistence of under- and over-nutrition within the same households (double burden households), (2) concentrated only on particular population subgroups (women) or small geographical areas (relatively affluent western Indonesia), or (3) failed to account for the influence of macro-level contextual factors. A large-scale population study covering the entire 17,000-island archipelago is, to our knowledge, non-existent as ‘there is little awareness of the double burden of malnutrition issues, be it in the government, the public or professional circles’ (Shrimpton & Rokx, 2013: 6; see also WHO, 2010).

Exploiting the fact that a large, nationally representative sample has recently become available, this paper aims to investigate the social determinants as well as the geographical variations of under- and over-nutrition among adults aged 15 years and older living in 440 districts in Indonesia. In particular, we are interested in understanding (1) the pattern of association between an individual׳s socio-economic position and his or her nutritional status; (2) the influence of contextual factors at the district level on one׳s probability of being under- or overweight; and (3) the geographical distribution of the risk of malnutrition within the archipelago after accounting for the effects of observable socio-demographic determinants. Because understanding *who* gets the diseases and *where* the diseases strike is imperative for tackling the double burden (UNSCN, 2006: 7), insights gained from this analysis are of high relevance for the formulation of evidence- or need-based intervention measures—especially for policy targeting in Indonesia as well as in other parts of the developing world.

## 2 Methods

### 2.1 Data

The data are drawn from the Riset Kesehatan Dasar (National Basic Health Research; henceforth ‘Riskesdas’) 2007. Managed by the Ministry of Health of the Republic of Indonesia, Riskesdas is the largest public health research initiative ever carried out in the country. The repeated cross-sectional study includes 987,205 individuals from 258,366 households residing in all 440 districts and is thus representative of the Indonesian population (Kemenkes, 2008). Its size and geographical coverage clearly distinguish Riskesdas from the Indonesia Family Life Survey (IFLS) dataset (30,000 individuals living in 260 districts) that was analysed in some earlier studies (Doak et al., 2005, Roemling and Qaim, 2013). Hence, in addition to the benefit of additional statistical power, Riskesdas also offers the opportunity for researchers to extend their inferences to the deprived and usually neglected islands of the archipelago (Sulawesi, Maluku, Halmahera, Nusa Tenggara and Papua). Informed consent was obtained prior to interview and participants׳ confidentiality was strictly protected. Further details regarding ethical and sampling procedures are available through Kemenkes (2008).

Included in the sample of this study are adults aged 15 and older. After excluding pregnant women and individuals of extreme height (less than 100 cm or more than 200 cm) or weight (less than 25 kg or more than 200 kg), the final sample size was 645,032 individuals. This corresponds to approximately 97% of all adults who participated in the Riskesdas 2007 study.

### 2.2 Variables

The dependent variable is adult nutritional status as indicated by body mass index (BMI). BMI is calculated by dividing an individual׳s weight (in kilograms) by his or her squared height (in metres); following the standard adopted by the government of Indonesia (Kemenkes, 2008), the individual is then classified as ‘underweight’ (BMI<18.5), ‘normal’ (18.5≤BMI<25), ‘overweight’ (25≤BMI<27), or ‘obese’ (BMI≥27). However, for the sake of computational feasibility as well as ease of understanding, we collapse the last two categories (see also Gurrici, Hartriyanti, Hautvast, and Deurenberg (1998) and WHO Expert Consultation (2004) for discussions regarding BMI cut-off points for obesity in the Indonesian context). Both the categorical representation of nutritional status and the continuous measure of BMI are used in the following statistical analysis.

The individual-level socio-economic explanatory variables of interest are education (indicator variables for primary education or less, secondary school, high school and college or more), employment status (dummy indicators for those who are not employed or in school) and per capita household expenditure (PCE) serving as a proxy for individual income. In Indonesia, as in many parts of developing world, the individual income measure is usually not available (reliable) due to the high prevalence of both self- and seasonal employment (60–70% in Indonesia; Nazara, 2010). The literature (Deaton and Zaidi, 2002, Howe et al., 2012) suggests that PCE is capable of delivering a good approximation for permanent income due to its insensitivity to intermittent income shock that is inherent in informal economy. Both the logarithmic and the quintile representations of PCE are used in the analysis.

At the district level, we include continuous measures of income inequality, level of economic development (median PCE in million Indonesian rupiah) and index of deprivation. Income inequality is measured using the Gini index on a scale of 0–1 and was derived from the PCE measure available in Survei Sosial Ekonomi Nasional (National Socio-economic Survey) 2007 dataset using the method described by Milanovic (1997). Subsequently, to aid with interpretation, this Gini index is multiplied by a factor of 10 before being used in any statistical modelling exercises. The deprivation index was calculated from the Potensi Desa (Village Census) 2008 dataset, covering all 75,410 villages across the archipelago. Factor loadings, proportion of shared variance as well as other statistics obtained during the derivation of the index are shown in Table 1. It is noteworthy, at this point, that the inclusion of measures of area-level economic development and facility deprivation alongside the income inequality variable allows researchers to separate the contextual effect of income inequality from the compositional effect of poverty (Subramanian & Kawachi, 2004).

**Table 1:** Exploratory factor analysis of district deprivation index.

Proportion of village without	Factor loading	Summary statistics	
Communication facilities	0.86	Explained variance	88%
Electricity	0.81	Cronbach׳s *α*	0.82
Street lighting	0.76	Eigenvalue	3.58
Healthcare facilities	0.75	KMO	0.80
TV signal coverage	0.73	*N*	454
Education facilities	0.65		
Entertainment facilities	0.30		

In the statistical models described next, we also control for survey respondent age group (15–24, 25–34, 35–44, 45–54, 55–64, or 65+), sex (dummy variable for female survey respondents), marital status (married, never married, divorced or widowed), self-report physical activity (indicator variable for those reporting inadequate physical activity according to the criteria set by Kemenkes (2008), urban/rural residential setting (dummy variable for urban residency), and number of household members. Continuous covariates are either centred to their respective grand means (log per capita household expenditure, Gini index) or to a representative value (household size of 3, deprivation index equals 0) so that the intercept can be meaningfully interpreted. Accordingly, for categorical variables the references are: married male aged 15–24 with primary school or less education, currently employed or in school, living in rural area with income at the poorest quintile and engaging in adequate physical activity.

### 2.3 Modelling techniques

In order to predict the nutritional status of individual *i* residing in district *j* with three possible nominal outcomes *s*={underweight, normal, overweight}, we specify the following generalised linear mixed model with logit link-function (Goldstein, 2011, Rabe-Hesketh and Skrondal, 2012):

$$\log\left[\mathrm{Pr}\left(y_{ij}=s\right)/\mathrm{Pr}\left(y_{ij}=\mathrm{normal}\right)\right]=X\beta^{\left(s\right)}+u_{j}^{\left(s\right)},\;s=\mathrm{underweight},\;\mathrm{overweight}.$$

In this specification, *X* is the matrix of explanatory variables at both individual and district level that also includes a constant term and cross-level interaction terms. The unknown parameter vector *β*^*(s)*^ captures the average effect of each explanatory variable on the probability of an adult being underweight or overweight relative to having a normal BMI. To facilitate interpretation, *β*^*(s)*^ is reported as a relative risk (odds) ratio. The $u_{j}^{\left(s\right)}$ is the contrast- and district-specific random effect that is assumed to be uncorrelated with *X* and is normally distributed with zero mean and variance to be estimated from the data. A parameter capturing the correlation (*ρ*) between random effects $u_{j}^{\left(s\right)}$ and $u_{j}^{\left(s+1\right)}$ is also obtainable from the model and is particularly useful for measuring the strength as well as the direction in which the risks of under- and over-nutrition covary within a single district. Such an interpretation has been used in some earlier studies in India (Subramanian and Smith, 2006, Subramanian et al., 2007); in fact, Corsi et al. (2011) have recently called for a wider use of this parameter to arrive at a formal way of assessing the existence of the double burden of malnutrition within a given geographical area. Furthermore, the fact that the estimated random effect *u*_*j*_^*(s)*^ is independent from the influence of observed socio-demographic characteristics is also helpful for the purpose of risk mapping or ranking (see Ackerson, Kawachi, Barbeau, and Subramanian (2008) for such an application to Indian data). It is important to note, however, that this model maintains the assumption of the independence of irrelevant alternatives (IIA), meaning that ‘adding or deleting alternatives does not affect the odds among the remaining alternatives’ (Long & Freese, 2006: 243). This should not be a particularly serious problem for the present study because the outcomes can plausibly be assumed to be distinct from one another (McFadden, 1973).

As an alternative to the multinomial outcome modelling exercise, which may suffer from a loss of information due to the arbitrariness of cut-off points, we also specify a quantile regression model (Koenker, 2005, Koenker and Hallock, 2001) that uses the continuous representation of BMI as the outcome variable. The model is given as follows:

$$Q_{q}\left(y_{i}\right)=X\beta^{\left(q\right)}+e_{i}^{\left(q\right)},\;q=0.05,\;0.10,\;\ldots \;,0.95.$$

In this specification, *Q*_*q*_(*y*_*i*_) denotes the *q*-th conditional quantile of BMI, *X* is the matrix of predictors with a constant term included, *β*^*(q)*^ is the vector of parameters capturing the effect of each explanatory variable on the *q*-th conditional quantile while holding all other covariates constant, and $e_{i}^{\left(q\right)}$ is the asymmetrically weighted absolute residual. Unlike in the linear model, neither specific distributional assumption nor homoscedasticity is assumed for the error term, making this non-parametric modelling technique relatively robust to the influence of outliers. The fact that one can obtain *β*^(*q*)^ estimates for a range of conditional quantiles and allow each predictor to have an impact on both the location and scale parameters of the model is useful for the purpose of understanding the heterogeneity in the relationship between BMI and its determinants. This possibility of obtaining a more complete picture of change in the conditional distribution of BMI is undoubtedly of particular interest from a public health perspective where monitoring both the upper and lower extremes of BMI is critical. It should be noted, however, that, unlike in mean regression, the conditional quantile is not generally equal to its unconditional (Firpo et al., 2009, Jolliffe, 2011). For purposes of computational feasibility with our large dataset, we address the clustering of individuals within districts by means of specifying a cluster-robust variance–covariance estimator (Machado et al., 2014, Santos Silva and Parente, 2013) instead of fitting a multilevel quantile regression model (Geraci, 2014, Geraci and Bottai, 2014).

## 3 Results

### 3.1 Descriptive and bivariate analysis

Table 2 presents descriptive statistics and measures of bivariate association between nutritional status and its predictors. BMI is approximately normally distributed (mean=22.05 kg/m^2^, median=21.52 kg/m^2^), albeit with some positive excess of kurtosis. The estimated national prevalence of underweight is 14.4% while that of overweight is 17.9%; despite our additional data cleaning procedure (Section 2.1), these figures remain very close to the official tabulation released by the Ministry of Health (14.8% and 19.1%, respectively; Kemenkes, 2008). These clearly show that, in 2007, one in three Indonesian adults was potentially suffering from nutritional problems and that the double burden of malnutrition in the country consisted relatively equally of both extremes of nutritional status.

**Table 2:** Sample description and bivariate analysis (*N*=645,032).

Variable	Descriptive statistic	Unadjusted odds ratio	
		Underweight	Overweight
**Nutritional status:**			
Body mass index	22.05±3.81		
Normal	67.7%		
Underweight	14.4%		
Overweight	17.9%		
**Age group:**			
Age 15–24	22.9%	1.00	1.00
Age 25–34	22.7%	0.40±0.01	2.72±0.04
Age 35–44	21.3%	0.33±0.01	4.26±0.07
Age 45–54	16.0%	0.45±0.01	4.39±0.09
Age 55–64	9.1%	0.80±0.02	3.44±0.08
Age 65+	8.0%	1.55±0.03	2.21±0.06
**Sex:**			
Male	48.8%	1.00	1.00
Female	51.2%	1.15±0.01	1.89±0.03
**Marital status:**			
Married	68.3%	1.00	1.00
Never married	23.4%	2.07±0.03	0.29±0.01
Divorced	1.8%	1.51±0.04	0.84±0.02
Widowed	6.5%	2.63±0.04	0.91±0.02
**Education:**			
Primary school or less	53.3%	1.00	1.00
Middle school	20.3%	0.92±0.01	0.92±0.01
High school	21.1%	0.68±0.01	1.22±0.02
College	5.3%	0.50±0.01	1.78±0.05
**Employment status:**			
In employment or schooling	88.9%	1.00	1.00
Unemployed	11.1%	2.07±0.03	0.65±0.01
**Physical activity:**			
Adequate physical activity	70.1%	1.00	1.00
Less physical activity	29.9%	1.47±0.02	1.18±0.02
**Residential setting:**			
Rural	62.6%	1.00	1.00
Urban	37.4%	0.95±0.02	1.78±0.04
**Household size and income:**			
Household size	4.59±1.90	1.00±0.00^a^	0.97±0.00
Log(PCE)	12.50±0.51	0.74±0.01	1.81±0.03
**District characteristics:**			
Median PCE (million Rupiah)	0.27±0.08	0.32±0.06	11.45±2.01
Deprivation (standardised)	−0.03±1.03	0.91±0.02	0.81±0.03
Inequality	0.25±0.04	1.02±0.03^a^	1.33±0.05

*Note:*

The prevalence of obesity as defined by BMI≥30 kg/m^2^ is 3.44%.

a *p*-Value>0.10; standard errors are adjusted for the clustering of individuals within 440 districts.

In the sample, sex is distributed equally; and the majority of survey respondents (92%) are of working age (15–64 years-old). About two-thirds of them are married; half have not completed the nine-year compulsory education; and most (70%) report adequate physical activity. Two-thirds of adults participating in the study live in a rural area; the average number of household members across residential settings is 4.6 persons per household; and the unemployment rate is at about 11%. Median monthly individual income is 258,421 Indonesian rupiah (USD 26), while the mean of the corresponding figure at the district level is IDR 265,638 (USD 27). Income inequality ranges from 0.13 (most egalitarian) to 0.40 (least egalitarian) with the mean equal to 0.25.

Bivariate association is presented in the last two columns of Table 2. As can be expected from a dataset that has large statistical power, nearly all parameters are precisely estimated. The odds of being both under- and overweight generally increase with being older (notably at age 65 and older), female, having inadequate physical activity, and living in a less egalitarian neighbourhood. Marriage, education, employment and income clearly protect Indonesians from being underweight, but they also increase the probability of being overweight. Larger household size is negatively associated with over-nutrition, but there is no statistically discernible effect on under-nutrition. Consistent with the pattern observed across the world, urban environments in Indonesia also seem to be obesogenic. A rather unexpected result, however, comes from the deprivation index. A priori, we would expect the coefficient for deprivation to have a positive sign in the underweight equation, yet at this early stage of analysis, our bivariate exploration suggests that the more deprived a region is, the smaller the odds of the residents being both over- *and* underweight. Whether this is simply an artefact of confounding is to be tested in the multivariate analysis presented next.

### 3.2 Multilevel multinomial logistic regression analysis

Having identified potential risk factors for under- and overweight through a simple bivariate procedure that does not take confounding into account, we now fit a series of multilevel multinomial logistic regression models to estimate the independent effect of each predictor on nutritional status (Table 3). The analysis is conducted in a stepwise manner: first, we fit an age–sex adjusted model (Null Model) before introducing the complete set of explanatory variables in the second model (Full Model 1); we further characterise the relationship between individual income and nutritional status by replacing the logarithmic parametrisation with indicators of income quintile (Full Model 2); finally, we consider the possibility of effect modification by interacting the female indicator with individual income and income inequality (Interaction Model). Goodness of fit is assessed by means of monitoring the Akaike/Bayesian information criterion statistic (AIC/BIC) such that models with smaller AIC/BIC are preferred over those with larger statistic.

**Table 3:** Adjusted odds ratio obtained from multilevel multinomial logistic models.

Predictors	Null Model		Full Model 1		Full Model 2		Interaction Model	
	Underweight	Overweight	Underweight	Overweight	Underweight	Overweight	Underweight	Overweight
Intercept	0.30±0.01	0.06±0.00	0.19±0.00	0.06±0.00	0.22±0.01	0.05±0.00	0.19±0.00	0.06±0.00
**Individual characteristics:**								
Age 25–34	0.39±0.00	2.77±0.04	0.57±0.01	1.99±0.03	0.57±0.01	1.99±0.03	0.57±0.01	1.99±0.03
Age 35–44	0.32±0.00	4.46±0.06	0.51±0.01	3.02±0.05	0.51±0.01	3.02±0.05	0.51±0.01	3.02±0.05
Age 45–54	0.43±0.01	4.64±0.06	0.66±0.01	3.15±0.05	0.66±0.01	3.17±0.05	0.66±0.01	3.15±0.05
Age 55–64	0.77±0.01	3.62±0.06	1.09±0.02	2.55±0.05	1.09±0.02	2.56±0.05	1.09±0.02	2.54±0.05
Age 65+	1.48±0.02	2.24±0.04	1.78±0.03	1.71±0.04	1.78±0.03	1.72±0.04	1.78±0.03	1.72±0.04
Female	1.12±0.01	1.95±0.01	1.11±0.01	2.00±0.02	1.11±0.01	2.00±0.02	1.11±0.01	2.10±0.02
Never married			1.79±0.02	0.49±0.01	1.79±0.02	0.49±0.01	1.79±0.02	0.49±0.01
Divorced			1.27±0.04	0.73±0.02	1.27±0.04	0.73±0.02	1.27±0.04	0.72±0.02
Widowed			1.24±0.02	0.84±0.01	1.25±0.02	0.84±0.01	1.24±0.02	0.84±0.01
Middle school			0.91±0.01	1.12±0.01	0.91±0.01	1.13±0.01	0.91±0.01	1.13±0.01
High school			0.79±0.01	1.16±0.01	0.78±0.01	1.18±0.01	0.79±0.01	1.16±0.01
College			0.72±0.02	1.23±0.02	0.71±0.02	1.27±0.02	0.72±0.02	1.21±0.02
Unemployed			1.10±0.01	0.99±0.02^a^	1.10±0.01	0.99±0.02^a^	1.09±0.01	0.99±0.02^a^
Less physical activity			1.20±0.01	1.10±0.01	1.19±0.01	1.11±0.01	1.19±0.01	1.10±0.01
Household size			0.98±0.00	1.03±0.00	0.98±0.00	1.03±0.00	0.98±0.00	1.03±0.00
Urban			1.01±0.01^a^	1.35±0.01	1.00±0.01^a^	1.36±0.01	1.01±0.01^a^	1.35±0.01
Log(PCE)			0.75±0.01	1.61±0.02			0.78±0.01	1.98±0.03
2nd PCE quintile					0.92±0.01	1.18±0.01		
3rd PCE quintile					0.89±0.01	1.34±0.02		
4th PCE quintile					0.80±0.01	1.51±0.02		
5th PCE quintile					0.72±0.01	1.81±0.02		
**District characteristics:**								
Median PCE			0.87±0.18^a^	1.57±0.33	0.34±0.07	7.34±1.53	0.87±0.18^a^	1.59±0.33
Deprivation			0.91±0.02	0.97±0.02^a^	0.92±0.02	0.97±0.02^a^	0.91±0.02	0.97±0.02^a^
Inequality			1.08±0.04	1.09±0.04	1.08±0.04	1.12±0.04	1.11±0.04	1.09±0.04
**Interaction terms:**								
Female×Log(PCE)							0.93±0.02	0.71±0.01
Female×inequality							0.94±0.02	0.99±0.02^a^
Between-district variance	0.12	0.20	0.10	0.11	0.10	0.12	0.10	0.11
Correlation between RE	−0.19		−0.19		−0.18		−0.19	
*N*	645,027		578,512		578,512		578,512	
AIC	1,018,045		891,342		891,623		890,755	
BIC	1,018,238		891,849		892,198		891,307	

Note:

a *p*-Value>0.10.

The age-sex adjusted model (Null Model) shows that, compared to their male counterparts, Indonesian women are more vulnerable to both under- and over-nutrition. Under-nutrition seems to be more prevalent in early adulthood (15–24 years old) and later life (65 years old and older) than in middle age. In contrast, the risk of over-nutrition seems to increase with age, peak at 45–54 years old, and then gradually decrease throughout the life course although the odds of being overweight are still about two times greater among the elderly than the youngest adults (15–24 years old). The random part of the model tells us that there seems to be a small negative correlation (*ρ*=−0.19) between the district-specific effects determining the probability of being under- or overweight. This means that places with high risk of under-nutrition tend to be the ones with low risk of over-nutrition; in other words, the double burden of malnutrition does *not* generally exist within the same districts in Indonesia. These age, sex and geographical patterns persist even when additional variables are introduced into subsequent models.

In fully specified models (Full Model 1, Full Model 2, Interaction Model), it is estimated that being underweight is negatively associated with being married, having a high education level, being employed, having a large household size, and having high income; yet these factors are also generally associated with greater odds of being overweight. The monotonicity of income effect is clearly demonstrated in Full Model 2, although a curvilinear parametrisation as introduced in Full Model 1 appears to be more parsimonious. This implies that, as of 2007, under-nutrition in Indonesia remains a ‘disease of poverty’, while over-nutrition is one of affluence. Having enough physical activity and living in an egalitarian area seems to protect Indonesians from both extremes of malnutrition, but area-level economic development only appears to aggravate the over-nutrition problem and does not seem to aid in alleviating under-nutrition even after controlling for facility deprivation and urban/rural residential location.

Finally, the Interaction Model tests whether women׳s nutritional vulnerability is modified by income level or income inequality. We found some evidence indicating that this is indeed the case. The model shows that as individual income increases, the nutritional gap between men and women narrows in both the underweight and overweight equations. The gap also diminishes as the level of income inequality increases in the underweight equation, but a similar effect is imprecisely estimated in the overweight equation. In essence, this tells us that the effect of income is more pronounced among women than men and that adults of both sexes are equally deprived when they live in less egalitarian environments. In all models, urban areas are consistently obesogenic while, rather paradoxically, facility deprivation remains negatively associated with under-nutrition. Ultimately, in order to ascertain whether these relationships are robust across disaggregations by sex and urban/rural location, we perform stratified analyses. Table 4 shows that these findings are indeed consistent.

**Table 4:** Adjusted odds ratio obtained from stratified models.

Predictors	Underweight				Overweight			
	Male	Female	Urban	Rural	Male	Female	Urban	Rural
Intercept	0.23±0.01	0.24±0.01	0.27±0.01	0.19±0.01	0.04±0.00	0.13±0.00	0.08±0.00	0.05±0.00
**Individual characteristics:**								
Age 25–34	0.53±0.01	0.61±0.01	0.56±0.01	0.57±0.01	1.87±0.05	1.92±0.04	2.02±0.08	1.99±0.04
Age 35–44	0.48±0.01	0.54±0.01	0.42±0.01	0.56±0.01	2.78±0.08	2.84±0.05	3.27±0.14	2.85±0.06
Age 45–54	0.58±0.02	0.75±0.02	0.45±0.02	0.78±0.02	3.00±0.09	2.90±0.06	3.73±0.17	2.78±0.06
Age 55–64	0.94±0.03	1.22±0.03	0.70±0.03	1.30±0.03	2.59±0.08	2.23±0.05	3.12±0.15	2.18±0.06
Age 65+	1.65±0.05	1.88±0.05	1.27±0.05	2.04±0.05	1.78±0.07	1.48±0.04	2.07±0.11	1.49±0.05
Female			1.02±0.02^a^	1.15±0.01			1.77±0.02	2.23±0.02
Never married	1.62±0.03	1.96±0.03	1.69±0.04	1.78±0.03	0.60±0.01	0.39±0.01	0.50±0.01	0.51±0.01
Divorced	1.30±0.07	1.28±0.04	1.25±0.07	1.29±0.05	0.76±0.05	0.70±0.02	0.74±0.03	0.71±0.03
Widowed	1.34±0.05	1.26±0.03	1.18±0.04	1.28±0.03	0.84±0.04	0.81±0.02	0.86±0.02	0.80±0.02
Middle school	0.91±0.01	0.90±0.01	0.88±0.02	0.93±0.01	1.26±0.02	1.06±0.01	1.08±0.02	1.13±0.02
High school	0.70±0.01	0.88±0.02	0.74±0.01	0.79±0.01	1.57±0.02	0.97±0.01	1.09±0.02	1.27±0.02
College	0.56±0.02	0.86±0.03	0.66±0.02	0.76±0.03	1.89±0.04	0.90±0.02	1.12±0.02	1.53±0.04
Unemployed	1.15±0.02	1.05±0.02	1.14±0.02	1.10±0.02	1.00±0.03^a^	1.01±0.03^a^	0.95±0.02	0.98±0.02^a^
Less physical activity	1.31±0.02	1.10±0.01	1.16±0.02	1.21±0.01	1.22±0.02	1.02±0.01	1.08±0.01	1.11±0.01
Household size	0.98±0.00	0.97±0.00	0.98±0.00	0.98±0.00	1.03±0.00	1.02±0.00	1.02±0.00	1.04±0.00
Urban	1.06±0.02	0.95±0.01			1.36±0.02	1.35±0.02		
2nd PCE quintile	0.93±0.02	0.92±0.01	0.92±0.02	0.92±0.01	1.20±0.02	1.16±0.02	1.17±0.03	1.19±0.02
3rd PCE quintile	0.89±0.02	0.88±0.01	0.88±0.02	0.88±0.01	1.39±0.03	1.30±0.02	1.32±0.03	1.36±0.02
4th PCE quintile	0.83±0.02	0.78±0.01	0.80±0.02	0.80±0.01	1.58±0.03	1.44±0.02	1.43±0.03	1.56±0.03
5th PCE quintile	0.76±0.02	0.69±0.01	0.71±0.02	0.73±0.01	2.00±0.04	1.64±0.03	1.65±0.04	1.93±0.03
**District characteristics:**								
Median PCE	0.38±0.09	0.29±0.06	0.24±0.06	0.33±0.09	8.09±1.81	6.41±1.43	4.48±1.06	12.31±3.44
Deprivation	0.88±0.02	0.96±0.02	0.86±0.03	0.93±0.02	1.03±0.02^a^	0.94±0.02	1.03±0.03^a^	0.95±0.02
Inequality	1.07±0.04^a^	1.08±0.04	1.11±0.05	1.05±0.05^a^	1.13±0.05	1.11±0.05	1.13±0.05	1.13±0.05
Between-district variance	0.11	0.11	0.10	0.12	0.12	0.13	0.11	0.14
Correlation between RE	−0.32	−0.18	−0.05	−0.24	−0.32	−0.18	−0.05	−0.24
*N*	283,218	304,643	213,942	364,570	283,218	304,643	213,942	364,570

Note:

a *p*-Value>0.10.

**Fig. 1:**
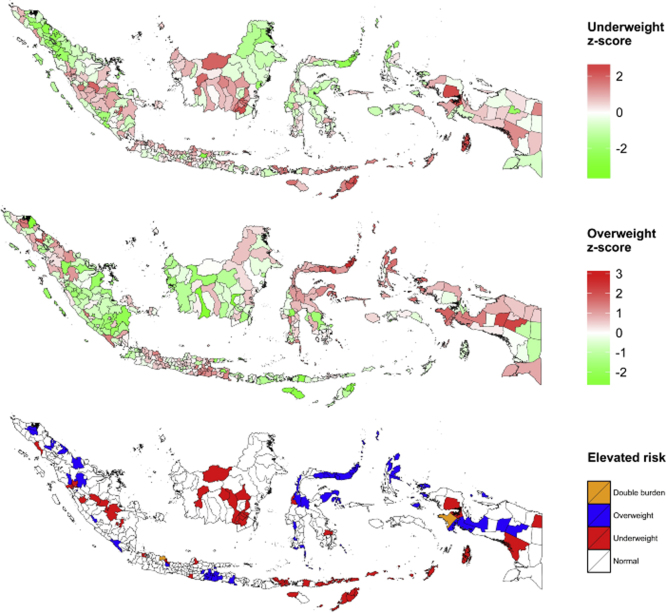
Spatial distribution of malnutrition across 440 districts in Indonesia.

Having investigated the determinants of nutritional status, we now attempt to understand the geographical distribution of the risk of malnutrition within the Indonesian archipelago by means of extracting the standardised random effects for each contrast (Ackerson et al., 2008) in the best fitting model (Interaction Model) and plotting them in the top and middle panels of Fig. 1. In this mapping exercise we manually impute the estimated random effects for two districts (Puncak Jaya and Pegunungan Bintang) with the value of their nearest neighbours (Jayawijaya and Yahukimo) because of a lack of individual income data in these districts. It is then evident from the maps that the risks of under- and over-nutrition are indeed spatially segregated across the islands in Indonesia. Clusters of areas with high under-nutrition vulnerability are observable in South Sumatra, Central and South Kalimantan (Borneo), Java (north coast), and Nusa Tenggara (Lesser Sunda) islands; areas particularly vulnerable to over-nutrition appear in North Sumatra, West and East Java, North and Central Sulawesi (Celebes), Halmahera, and Papua. Further, in the bottom panel we identify areas with elevated risk of dual malnutrition (*Z*-score>1). Only two out of 440 districts are categorised as double burden districts (Indramayu in West Java and Fak-Fak in West Papua); the number of districts classified as underweight and overweight is 54 and 66, respectively. Finally, Table 5 presents the top 10 most nutritionally vulnerable districts. It is apparent at this point that if evidence- or need-based interventions are to be prescribed, then the islands of Nusa Tenggara (containing four of the 10 districts most vulnerable to under-nutrition) and Sulawesi (containing eight of the 10 districts most vulnerable to over-nutrition) must be the primary targets.

**Table 5:** Top 10 most nutritionally vulnerable districts.

Under-nutrition			Over-nutrition		
Rank	District	Island	Rank	District	Island
1	Belu	Nusa Tenggara	1	Kota Tomohon	Sulawesi
2	Rote Ndao	Nusa Tenggara	2	Kota Bitung	Sulawesi
3	Kepulauan Aru	Papua	3	Minahasa Selatan	Sulawesi
4	Teluk Bintuni	Papua	4	Minahasa	Sulawesi
5	Banjar	Kalimantan	5	Jayawijaya	Papua
6	Timor Tengah Utara	Nusa Tenggara	6	Bone Bolango	Sulawesi
7	Hulu Sungai Utara	Kalimantan	7	Kota Manado	Sulawesi
8	Timor Tengah Selatan	Nusa Tenggara	8	Minahasa Utara	Sulawesi
9	Kapuas Hulu	Kalimantan	9	Karo	Sumatra
10	Tebo	Sumatra	10	Kota Gorontalo	Sulawesi

### 3.3 Quantile regression analysis

**Fig. 2:**
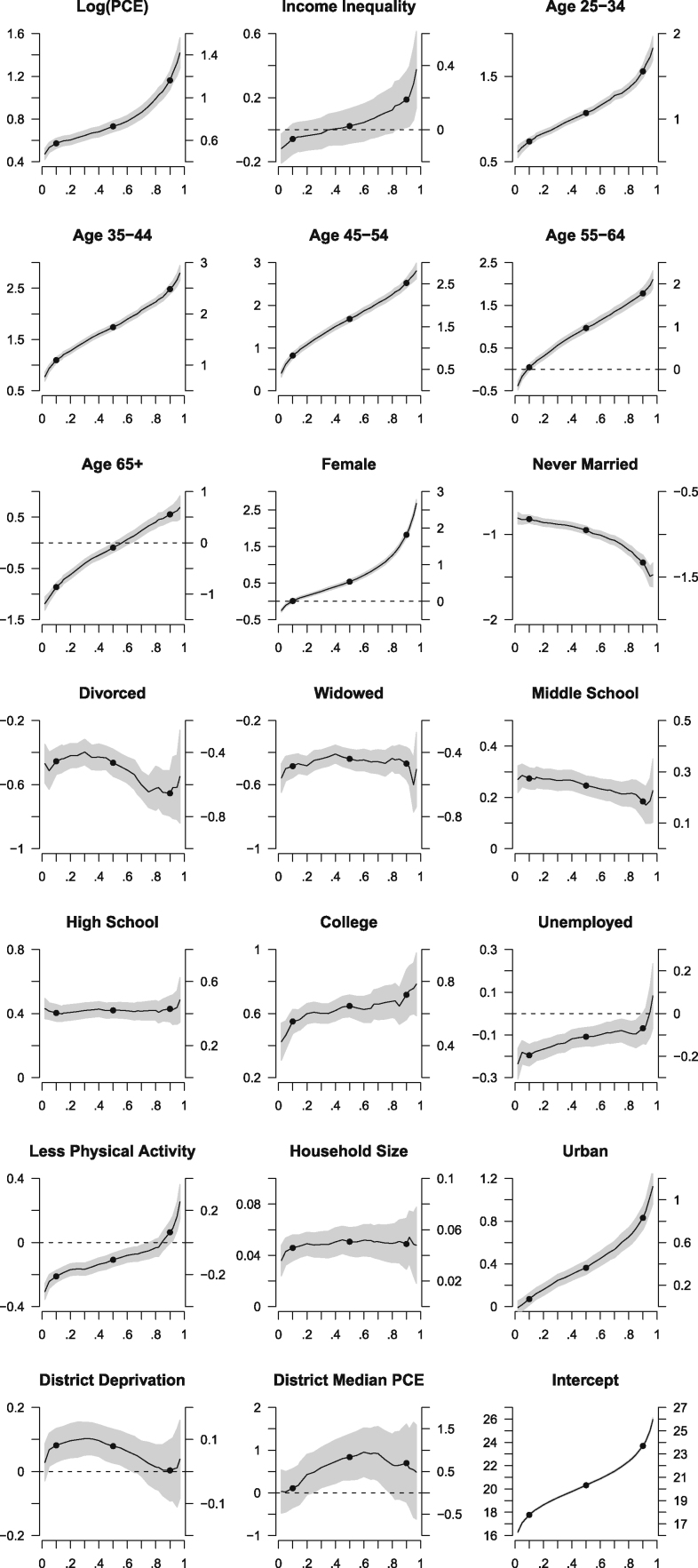
Quantile regression estimates (BMI quantiles in *X*-axis; *β* estimates in *Y*-axis).

The previous modelling exercises have implicitly assumed that the relationship between nutritional status and its predictors is homogeneous along the continuum of BMI. In this section, we relax this assumption by allowing each predictor to have an impact on both the location and the scale of conditional BMI distribution. The result of fitting a quantile regression model with the Full Model 1 specification is presented in Fig. 2. In the figure, the *X*-axis represents the conditional quantile of BMI, while the *Y*-axis indicates the estimated regression coefficient; a bold black line shows the independent effect of each explanatory variable on the respective conditional quantile with its associated 95% point-wise confidence interval shown in grey shade; the three solid black circles represent the conditionally underweight (the 0.1th quantile), normal (the 0.5th quantile) and overweight (the 0.9th quantile). The goal of this modelling exercise is to find out for whom the effect of each covariate is particularly relevant. A flat line means that the effect is equal for all individuals, irrespective of their nutritional status. A monotonically increasing or decreasing line indicates that the effect becomes gradually more pronounced in one extreme of nutritional status. A U-shaped line suggests that the effect is different between individuals with BMIs in the normal range and those at both extremes of the nutritional spectrum. Finally, any line crossing the zero *Y*-axis shows that there is a divergence in the direction (a positive-to-negative reversal, or vice versa) of an effect.

As shown in Fig. 2, being married, having a high education level, being employed and having one additional household member are associated with a constant positive increase of BMI. In contrast, the effects of income, age and urban environment on BMI are monotonically positive with magnitudes that become increasingly stronger as one moves from the underweight to the overweight sub-population. An exception, though, is the oldest age group (65 years old and older). Among the underweight, later life is associated with a lower BMI, while among the overweight, it is associated with a higher BMI; this is, however, of little consequence for normal individuals. A roughly similar pattern is observable for sex, physical activity and income inequality. This means that being female, having inadequate physical activity, being in the oldest age group and living in a less egalitarian area are especially detrimental for the under- and overweight sub-populations. U-shaped relationships are observable for the effects of deprivation and area-level economic development. This suggests that a positive change in these variables is associated with a higher BMI; it is, however, only statistically significant among individuals with BMIs in the normal range. The relatively straight intercept estimates show that BMI is approximately normally distributed, which indeed confirms the result of our earlier descriptive analysis (Section 3.1). Overall, regardless of the differences in modelling assumptions, the picture obtained from the quantile regression model largely mirrors that of the multilevel multinomial logistic models.

## 4 Discussion and conclusion

Analysing a nationally representative dataset, this paper investigates the social determinants as well as the geographical variations of the double burden of malnutrition in 440 districts in Indonesia. The main objectives of this research are to study (1) how individuals׳ socio-economic positions relate to nutritional status, (2) how contextual factors at the district level influence individuals׳ nutritional status, and (3) how the risks of under- and over-nutrition are distributed around the Indonesian archipelago after adjusting for the effects of observable socio-demographic determinants.

We found that, in 2007, the prevalence of under- and overweight was 14.4% and 17.9%, respectively. These figures indicate that one in three Indonesian adults faces a potential nutritional problem and that the double burden of malnutrition is shared roughly equally by both under- and over-nutrition problems. We found that education, employment, and income protect Indonesians from under-nutrition but that they also increase the probability of being overweight. Individual income as measured using per capita household expenditure seems to exhibit a monotonically decreasing and increasing effect on the likelihood of being under- and overweight, respectively. This suggests that under-nutrition in Indonesia remains a disease of the poor while over-nutrition is one of the affluent, a finding consistent with the general trend observed in other low and lower-middle income countries but not among upper-middle and high income countries (Jolliffe, 2011, Popkin, 2001, Subramanian et al., 2009).

The risk of under- and over-nutrition seems to be spatially clustered within the islands of Indonesia. Clusters of districts with high under-nutrition vulnerability are located in South Sumatra, Central and South Kalimantan, Java (north coast), and Nusa Tenggara islands; susceptibility to over-nutrition is observed particularly in North Sumatra, West and East Java, North and Central Sulawesi, Halmahera, and Papua. We found little evidence to suggest that the double burden of malnutrition exists within the same districts in Indonesia. Areas with high risk of under-nutrition tend to be the ones with low risk of over-nutrition; in fact, only Indramayu district in West Java and Fak-Fak district in West Papua are identified as double burden districts. To some extent, this is perhaps a relief from the point of view of policy-makers, for whom the burden of under- and over-nutrition coexisting within the same districts might have presented a somewhat difficult situation. As previous research has already pointed out, though, despite appearing to be a transitory phenomenon, the double burden of malnutrition does indeed appear in a significant portion of individual Indonesian households (Doak et al., 2005, Oddo et al., 2012, Roemling and Qaim, 2013, Vaezghasemi et al., 2014).

While finding little evidence for the presence of double burden districts, we have identified the existence of ‘doubly vulnerable’ population sub-groups. Our analysis shows that the elderly, women, individuals engaging in insufficient physical activity, and individuals living in highly unequal districts are vulnerable to both under- and over-nutrition problems. We suspect that, for the elderly, this is due to the changes in metabolic function and lifestyle as well as the psychological challenges associated with ageing (Hickson, 2006). For women, the double vulnerability seems to be consistent with explanations provided by the biological, social and cultural aspects of malnutrition (Brown and Konner, 1987, Delisle, 2008). On the one hand, some suggest that women׳s propensity to obesity is driven by the difficulty of maintaining a healthy weight after the high nutritional requirements of childbearing (pregnancy and lactation) subside; in some parts of the developing world, the tendency to obesity is further shaped by the ideal body image maintained by society (fatness as a symbol of maternity, nurturance and affluence). On the other hand, researchers also document that women in some poor societies are often subjected to gender discrimination in intra-household food allocation, hence posing a greater risk of under-nutrition (Frongillo and Bégin, 1993, Molini and Nubé, 2007, Thomas, 1990).

Regarding the adverse effect of income inequality on nutritional status, Subramanian et al. (2007) suggest in their study of Indian society that income inequality can be a marker of both resource maldistribution and inefficient public policy. It is likely that unequal areas are the places where the privileged over-consume while the underprivileged face food insecurity. Equally likely is that, due to the low social cohesion as well as other negative externalities associated with a highly skewed income distribution, public policy in a less egalitarian society is prone to manipulation by vested interests, resulting in poor provision of the amenities that are vital for combating malnutrition.

In our research, we also found a paradoxical protective effect of facility deprivation on under-nutrition. We initially suspected this to be an artefact of confounding, but it remains unresolved even after fitting multivariate models. While puzzling, this is not an isolated observation (Thomas and Strauss, 1992, Wolff and Maliki, 2008). Perhaps this is attributable to the endogenous, non-random spatial distribution of government programs as a result of the historical priority on placing health facilities and interventions in less healthy areas (Pitt, Rosenzweig, & Gibbons, 1995). Unfortunately, this puzzle cannot simply be addressed using the cross-sectional data we have at hand; it may therefore be pursued further in future research.

Other limitations of this study must now be acknowledged. The cross-sectional data that we have do not permit us to incorporate the temporal dimension into our analysis. As a consequence, this study only provides a snapshot capturing the determinants and geographical variations of the double burden of malnutrition in Indonesia in the year 2007. It is known that the burden of obesity gradually shifts to the poor as a nation progresses economically (Brown and Konner, 1987, Popkin, 1998). Whether such a shift has begun to occur in Indonesia is indeed an interesting subject to study, but carrying out the relevant research obviously necessitates the availability of newer data. Another limitation is that the statistical models fitted in this study did not explicitly account for spatial-contextual autocorrelation which may, to some extent, affect the precision as well as the smoothness of the estimated risks. The importance of undertaking such an endeavour cannot be underestimated, but it clearly deserves its own avenue in the vast literature of spatial epidemiology.

Despite these limitations, this study does, however, contribute to the literature in several ways. This study is among the few to consider the double burden of malnutrition in Indonesia from the perspective of the general population. As noted earlier, all existing studies have focused rather specifically on Indonesian women (Winkvist et al., 2000) or households (Doak et al., 2005, Oddo et al., 2012, Roemling and Qaim, 2013, Vaezghasemi et al., 2014). This study also adds to the literature by showing that the influence of contextual macro-economic conditions (income inequality and level of economic development) is not negligible with regard to the nutritional well-being of individuals (Block et al., 2004). In addition, this study provides the literature with a principled characterisation of the spatial distribution of nutritional vulnerability within the 17,000-island Indonesian archipelago which, we believe, is indispensable for the purpose of policy targeting. Of course, in the absence of good data, this study would not have been able to offer the present analysis.

If any policy implications for dealing with the double burden of malnutrition are to be suggested from the findings of this study, then they should include the following points. Raising the overall level of the socio-economic status of the population through education, employment, and income-enhancing opportunities can help to improve purchasing power, which, in turn, enables individuals to afford enough food to fulfil their needs. That alone, however, is not sufficient; we have already seen that the risk of over-nutrition also increases with every improvement in socio-economic conditions. Therefore, there is a need for a wider public educational campaign that promotes behavioural changes especially in, but not limited to, the spheres of physical activity, dietary pattern and gender equality (Roemling & Qaim, 2012). Furthermore, the need for better nutritional education (Webb & Block, 2004) in academic curricula cannot be overstated as it has become apparent that, at least in our models, more schooling is not always correlated with better nutritional status. Better nutritional education, of course, will not only facilitate behaviour change but also help to shape a healthier body image in society. Simultaneously, as it has been projected that by the year 2030 more people in developing countries will live in cities than in rural areas (Cohen, 2006), the obesogenic urban environment must also be addressed. A recent assessment of Indonesia׳s built environment indicates an environment ‘that is fairly unfriendly to pedestrian physical activity with limited access to healthy foods’ (Shrimpton & Rokx, 2013: 3). This hints that improvement in nutritional health can also be achieved through the provision of a healthier urban planning initiative.

Furthermore, as much as nutritional well-being is determined by genetic predisposition and individual behaviour, it is also a matter of social justice. While the effects of inequality may appear relatively minor, they affect millions of Indonesians. An economic policy that promotes equity and quality of development as opposed to one that emphasises growth per se is much desired. This entails the aim not only to narrow the gap between the haves and the have-nots within a region, but also to distribute the fruit of development fairly between regions. As shown in the nutritional vulnerability map (Fig. 1), it is no coincidence that places with high risk of under-nutrition tend to be the ones that are difficult to access and that have an inefficient distribution system and low market penetration. Indonesians living in these remote areas, no matter how much spending power they have, still find it difficult to achieve diversified, nutritionally balanced diets relative to those living in other parts of the archipelago. Perhaps it is not too late to remind ourselves that an efficiently functioning market and distribution system constitutes a necessary condition for a nation׳s nutritional well-being. Lastly, it is also worth noting that, if any interventions are to be initiated, then islands in east Indonesia should now clearly be the top priority for policy-makers.

## Author contributions

WH conceived the study, analysed and interpreted the data, and wrote and edited the manuscript. GT contributed to the conceptualisation of the study, interpretation of the results, and editing of the manuscript. The authors had no conflicts of interest to declare.
